# A poor diet quality is associated with more gas-related symptoms and a decreased quality of life in French adults

**DOI:** 10.1017/S0007114522001593

**Published:** 2023-02-28

**Authors:** Soline Chaumont, Laurent Quinquis, Bénédicte Monnerie, Chloé Six, Pascale Hébel, Olivier Chassany, Martin Duracinsky, Boris Le Nevé

**Affiliations:** 1 Danone Nutricia Research, Palaiseau, France; 2 Excelya, Boulogne-Billancourt, France; 3 CREDOC, Centre de Recherche pour l’Etude et l’Observation des Conditions de vie, Paris, France; 4 Patient-Reported Outcomes Research Unit, Unité de Recherche Clinique en Economie de la Santé (URC-ECO), Hôpital Hôtel-Dieu, AP-HP, Université de Paris, Paris, France

**Keywords:** Dietary patterns, General population, Digestive symptoms, Diet quality index, Intestinal Gas Questionnaire

## Abstract

This study evaluated the association between dietary patterns, gas-related symptoms (GRS) and their impact on quality of life (QoL) in a representative sample (*n* 936) of the French adult population. During the 2018–2019 ‘Comportements et Consommations Alimentaires en France’ survey (Behaviors and Food Consumption in France), online evaluation of GRS in adult participants was performed using the validated Intestinal Gas Questionnaire (IGQ), which captures the perception of GRS and their impact on QoL via six symptom dimensions scores (range 0–100; 100 = worse) and a global score (mean of the sum of the six symptom dimensions scores). Socio-demographics, lifestyle parameters and dietary habits (7-d e-food diary) were also collected online. Quality of diet was determined using the Nutrient-Rich Food 9.3 (NRF9.3) score (range 0–900; 900 = best). Univariate and multivariate linear regression models were applied to identify factors associated with IGQ global score. K-means was used to identify clusters of subjects based on their dietary records. Data from 936 adults who completed both the IGQ and the food diary showed a mean IGQ global score of 11·9 (sd 11·2). Younger age and female sex were associated with a higher IGQ global score. Only 7 % of subjects reported no symptom at all and nearly 30 % of study participants reported a high impact of GRS on their QoL. Two dietary clusters were identified: cluster 1, characterised by a higher consumption of fruits and vegetables, lower sugars intake and higher NRF9.3 score and cluster 2, characterised by higher intake of sugars, lower intake in dietary fibres and lower NRF9.3 score. The IGQ global score was lower in cluster 1 and higher in cluster 2 *v.* the total sample average (*P* < 0·001). The prevalence of GRS in the French adult population is high and is associated with impaired QoL and dietary patterns. A change in food habits towards healthier patterns could help reducing the burden of GRS.

Gas-related symptoms (GRS) such as bloating, borborygmi or flatulence are common complaints among subjects from the general population^(1–5)^. However, available data on the prevalence of GRS in the general population are limited and often obtained using non-validated tools. Except for studies in patients with irritable bowel syndrome (IBS) and other disorders of gut–brain interactions (DGBI)^(6–8)^, the impact of GRS on quality of life (QoL) in the general population has received little attention.

The Intestinal Gas Questionnaire (IGQ) is the first validated tool assessing the severity of six GRS and their impact on daily life in the general population. It has been developed and psychometrically validated in both patients with IBS and subjects from the general population, simultaneously in three countries (UK, Spain, France)^(9)^.

Patients with IBS frequently report that food can elicit or worsen their digestive symptoms^(10,11)^, and fermentable fibres are commonly associated with GRS like bloating or flatulence. High-residue diet challenges (e.g. bean meal) have thus been shown to increase intestinal gas production (volume, frequency of evacuations) and abdominal symptoms in patients with DGBI complaining of GRS^(12–15)^. Moreover, dietary restriction strategies like the low fermentable oligo-, di-, monosaccharides and polyols (FODMAP) diet are currently used to relieve IBS symptoms^(16–18)^.

The associations between GRS and other factors such as socio-demographics, lifestyle and diet remain poorly explored in the general population. Available findings report a higher prevalence of GRS in females and a lower prevalence in older individuals (> 65 years old)^(1,3)^. Higher prevalence of GRS is associated with a lower health-related QoL and a negative impact on emotional, social and physical well-being^(3,4)^. The potential role of lifestyle and diet has not been investigated in epidemiological studies, even though affected individuals consider lifestyle and food as the main factors responsible for their GRS^(4)^. In a retrospective infodemiology study recently conducted in French-speaking online forums, food and psychological factors (e.g. stress) were perceived as the main causes of digestive discomfort by web users^(19)^.

Capturing overall dietary patterns and not only using simple nutrient analysis is important to reflect the complex exposure of interacting food components consumed daily in different combinations^(20)^. Recently, dietary pattern analysis has emerged as an approach to explore the relationship between diet and health in nutritional epidemiology^(21)^. Diet quality indexes such as the Nutrient-Rich Food 9.3 (NRF9.3) score^(22)^, the Healthy Eating Index^(23)^ or the Probability of Adequate Nutrient Intake (PANDiet)^(24)^ were developed upon national or international nutrition guidelines and are validated and recognised tools to interpret dietary records, allowing results comparison between different cohorts/data sets. However, such indexes are only providing a very broad level of information, that is, overall quality of the diet. In complement, exploratory/unsupervised methods such as dietary clustering allow to generate more detailed insights from collected dietary data (nutrients and food group patterns)^(25)^.

Therefore, the aims of this study were to evaluate for the first time (i) the prevalence of GRS and their impact on QoL in a representative sample of the French adult population^(9)^ and (ii) the association between GRS, socio-demographics, lifestyle and dietary patterns.

## Materials and methods

### Ethical considerations

This epidemiological survey was approved by the independent scientific council at Crédoc and did not require a submission to an ethical committee, according to French regulation. The addition of the IGQ did not require a submission to an ethical committee. The Crédoc conducted data collection from the Comportements et Consommations Alimentaires en France (CCAF) online survey and owns the data. This survey was conducted according to the guidelines laid down in the Declaration of Helsinki, the General Data Protection Regulation, and all procedures involving human subjects were approved by the French Commission Nationale de l’Informatique et des Libertés. Informed consent was obtained from all subjects that participated in the 2018–2019 CCAF survey, including consent for publication of anonymised data. ‘LE TERRAIN company’ formally undertakes to preserve the anonymity of participants in the CCAF study, to secure the information specific to each participant and never to disclose their identity for commercial or free purposes.

### Population

Cross-sectional CCAF surveys (Behaviors and Food Consumption in France) are periodically conducted by the Crédoc in representative samples of the French population to collect data on anthropometry, socio-economic status, lifestyle and diet. They constitute a database to explore specific aspects of nutrition^(26,27)^. The general methodology of CCAF surveys has been published previously^(26)^. Briefly, the Crédoc conducted phone interviews to recruit participants for the 2018–2019 CCAF online survey that was carried out between October 2018 and July 2019 in a nationally representative sample of 2947 participants. Informed consent was provided online via the CCAF platform. Included participants then received a link towards an 7-d e-food diary. Socio-demographic and lifestyle questionnaires were displayed online on the last day of completion of the 7-d e-food diary. Questions included age, sex, socio-economic status (education, income, profession, composition of household, etc.), geographical region, town size, self-reported height and weight and time spent on physical activities or sedentary behaviour (e.g. daily screen watching).

To control for seasonal differences in food consumption, the survey was carried out in four successive recruitment waves: November–December, January–March, April–mid-June and mid-June–July during each of which approximately a quarter of the participants were included. For each recruitment wave, participants were selected according to the quota sampling method with age, sex, socio-economic status (occupation of the head of household as classified by the French National Institute of Statistics), geographical region, town size and household size, taken into consideration.

**Fig. 1.**
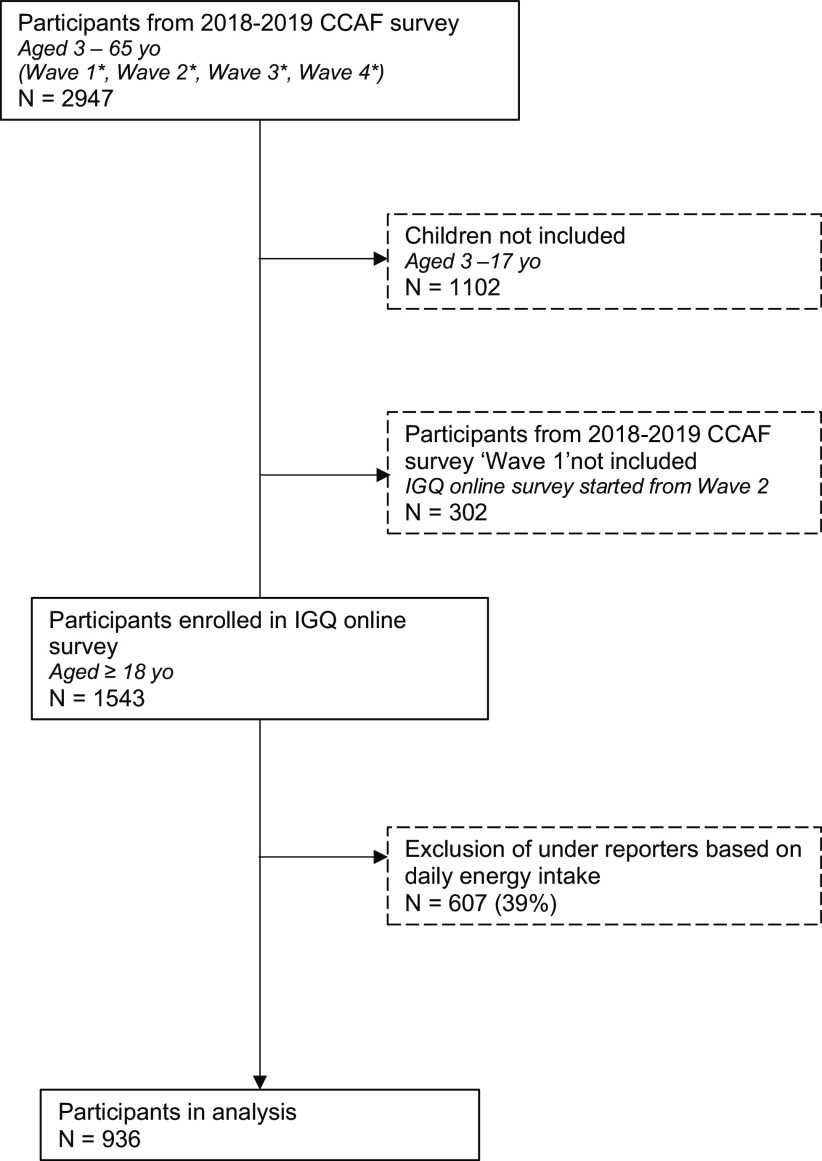
Flow chart of study participants. yo: years old; *Wave 1: October 2018–November 2018, Wave 2: December 2018–February 2019, Wave 3: February 2019–April 2019, Wave 4: April 2019–August 2019.

As the IGQ was not available before December 2018, evaluation of GRS in adult participants from CCAF survey could only be performed on recruitment waves 2–4 between January 2019 and July 2019. Briefly, IGQ was displayed online along the CCAF socio-demographic and lifestyle questionnaires on the last day of completion of the 7-d e-food diary. Immediately after completing the IGQ, participants were asked whether they took some actions in the past 7 d to relieve any GRS from a list of solutions. Pre-listed solutions included consulting a physician, taking drugs, consuming fermented food products (e.g. yogurt), consuming supplements (homeopathy, phytotherapy, probiotics) or starting a specific diet (e.g. low FODMAP). Participants could also report other non-pre-listed solutions or declare having taken no action. Participant’s inclusion details as well as timeline are described in Fig. 1.

### BMI calculation

The body adiposity status was calculated using the BMI as weight/height^2^ (kg/m^2^). In adults, BMI values are defined as underweight with BMI < 18·5 kg/m^2^, normal within the range 18·5–24·9 kg/m^2^, overweight within the range 25–29·9 kg/m^2^ and obesity with BMI ≥ 30 kg/m^2(28)^.

### Determination of physical activity indicator

The time spent watching various screens as well as the time spent on various physical activities was reported with the Recent Physical Activity Questionnaire from INCA3 study^(29)^. Terciles of this indicator were used to classify participants.

### Diet quality

To assess the relationship between GRS and dietary intake, the overall diet quality was assessed using the NRF9.3. In short, the NRF9.3 nutrient profiling model is based on the difference between the sum of daily values (%DV) percentage for nine nutrients for which intake is recommended, and the sum of daily values percentage for three nutrients for which intake is to be limited. Higher NRF9.3 scores indicate better nutrient adequacy, and a maximum possible score of 900 suggests a diet in which intake per given amount of energy is above the recommended daily values (RDV) for the nine qualifying nutrients and are below the RDV for the three disqualifying nutrients^(30)^.

### Gas-related symptoms measure

The severity of GRS and their impact on daily life was measured using the IGQ. IGQ has been developed and psychometrically validated simultaneously in three countries (UK, Spain, France). The questionnaire consists of seventeen items: seven items measuring the severity of six GRS with a 24-h recall period (bloating, flatulence, belching, bad breath, stomach rumbling and difficult gas evacuation) and ten items assessing the impact of these symptoms on different aspects of QoL with a 7-d recall period. The response options for items range either on a numeric scale from 0 to 10 or on a 0 to 4 Likert scale. The score of each of the six symptom dimensions and the global score (mean of the sum of the symptom dimensions scores) range from 0 to 100 (worse)^(9,31)^.

To identify individuals mostly affected by their symptoms, we defined a threshold based on the impact of GRS on QoL. Subjects having answered at least at mid-scale to one question among the ten QoL impact items were defined as significantly impacted (i.e. having answered at least five on numeric scale items and at least ‘moderate’ for Likert scale items).

### Dietary intake data

Dietary intake was assessed based on an 7-d e-food diary. The participants reported the types and amounts of all foods and beverages consumed during 7 d. For each eating occasion during the week, all intakes of foods and beverages had to be reported. The e-food diary had separate pages for the different daily meals, including main meals (‘breakfast’, ‘lunch’ and ‘dinner’) and the various possible snacking occasions in between main meals. To facilitate portion reporting, participants were provided with the validated SUVIMAX portion size atlas (SUVIMAX 2000) describing various commonly consumed foods and beverages in seven different portion sizes.

The energy and nutrient contents of consumed foods and drinks were obtained from the national French Food Composition Table (CIQUAL 2013, https://ciqual.anses.fr/) updated in 2018. Intakes were computed for the whole day and for individual eating occasions (main meals and snacks). The circumstances of intake were reported by the participants, including time of day, day of the week, location and context of consumption (e.g. alone, seating, with friends, etc.).

### Statistical analysis

The analysis set comprises participants included during the three last recruitment waves of the 2018–2019 CCAF survey who completed both the IGQ and the 7-d e-food diary. The energy intake reported by the participants was compared with the estimated energy requirements according to Schofield’s equation^(32)^, a method commonly used to identify under-reporters. Under-reporters were excluded (declared energy intake 0·95 times the metabolic rate). This cut-off value was chosen on the basis of Black’s practical guide that proposed a lower 95 % CI of 1·05 for moderately active individuals^(33)^.

Calibration on margins (‘icarus’ R package) was used to redress the sample and weighted IGQ global score and symptom dimensions scores by using the region, living area, sex, age, occupation and type of household as weighting factors. All results displayed in the paper are reweighted estimators from the survey sampling calibration.

Lifestyle and socio-demographic characteristics, GRS and dietary intake were described using mean values and standard deviations for quantitative data and frequency (percentage) for qualitative data.

Clustering analysis was performed in two steps to identify patterns from the dietary data. We first computed a principal component analysis on all food categories (thirty-eight groups) entered in grams and standardised to a mean of 0 and an sd of 1 to ensure that quantities consumed were comparable across different categories. Then we applied the K-means clustering method on the two first principal component analysis dimensions. The number of clusters was chosen based on the Elbow, Silhouette and Gap statistics and a pragmatic balance between the number of participants within each cluster and the ease in interpreting the results. To assess over/under-representation of factors within each cluster as compared with the overall sample, the V-Test was performed using the ‘catdes function’ (FactoMineR R package).

Factors associated with the IGQ global score were identified using univariate and multivariate linear regression models. First, we ran univariate linear models with IGQ global score as a factor to explain each lifestyle, socio-demographic and dietary parameters as explicative factors. All factors having a *P*-value lower than 0·2 in the univariate model were selected for the multivariate linear regression model. The final model to explain IGQ global score variations was obtained using both backward and forward methods in addition with the Akaike information criterion^(34)^. The 0·1 *P*-value threshold was used to select the factors staying in the multivariate model at each step.

The R software (https://www.r-project.org/) was used for all statistical analyses and for database management. A margin of error of 5 % was used for all individual two-sided statistical tests. No adjustment for multiplicity was applied, so all significant results must be considered as exploratory. As this is the first use of IGQ in combination with socio-demographic, lifestyle and dietary parameters in a nationally representative sample, no *a priori* hypothesis was made regarding the prevalence of GRS in French adults, and we did not *a priori* consider the power of the study.

## Results

### Socio-demographic and lifestyle characteristics

After excluding children (*n* 1102) and participants from CCAF WAVE 1 (*n* 302) as the IGQ online version was not available at that time, 1543 adult participants from the original cohort of 2947 individuals were enrolled in the IGQ online survey. Exclusion of under-reporters based on daily energy intake (*n* 607) yielded an analysis set of 936 participants. The flow chart of study participants is detailed in Fig. 1.

Participant characteristics are shown in Table 1. Fifty-two percents were women, 24·9 % had over 65 years, 40·5 % were couple without children and 29·5 % were living in an area with more than 100 000 inhabitants. Most participants reported being non-smokers (81·1 %), without sleep disturbances (88·1 %) and working as employees (39·7 %). According to BMI data, 57·5 % had normal weight, 29·7 % were overweight and 9·5 % were obese with 19 % of the participants following a diet. The mean NRF9.3 score was 672·2 (sd 96·3).

**Table 1.** Summary of population characteristics (*n* 936) (Numbers and percentages)

		*n*	Weighted, *n*	%
Sex	Female	572	489·5	52·3
Age (years)	18–24	99	116·2	12·4
	25–34	181	119·7	12·8
	35–44	125	140·2	15·0
	45–54	180	177·2	18·9
	55–64	194	149·7	16·0
	65+	157	233·1	24·9
Occupation	Farmer, artisan, trader, business owner	29	75·9	8·1
	Executive, Senior Intellectual, Liberal	358	114·2	12·2
	Intermediate occupation	167	190·9	20·4
	Employee	248	371·5	39·7
	Worker	38	60·1	6·4
	Never employed/inactive	96	123·4	13·2
Living area	Rural (less than 2000 inhabitants)	190	193·8	20·7
	2000 to less than 20 000 inhabitants	156	189·1	20·2
	20 000 to less than 100 000 inhabitants	121	126·4	13·5
	100 000 inhabitants or more	313	276·1	29·5
	Paris	156	150·7	16·1
Type of household	Single	210	191·9	20·5
	Couple without children	302	379·1	40·5
	Couple with child(ren)	337	254·6	27·2
	Single parent family	40	84·3	9·0
	Other	47	26·2	2·8
Income	Less than 1700 €/1967 USD	136	172·2	18·4
	From 1700 €/1967 USD to less than 3000 €/3471 USD	206	217·2	23·2
	From 3000 €/3471 USD to 4500 €/5206 USD	230	228·8	24·4
	More than 4500 €/5206 USD	141	93·6	10·0
	Refusal to disclose	223	224·2	24·0
Region	Paris	178	174·1	18·6
	West Paris	97	86·1	9·2
	East Paris	73	71·1	7·6
	North	35	58·0	6·2
	West	136	129·2	13·8
	East	67	79·6	8·5
	Southwest	116	106·7	11·4
	Centre	132	115·1	12·3
	Mediterranean	102	116·1	12·4
Smoking	Yes	165	168·2	18·0
	No	763	759·3	81·1
	Missing	8	8·5	0·9
BMI	Underweight	34	31·3	3·3
	Normal	597	538.	57·5
	Overweight	230	277·7	29·7
	Obese	75	89·0	9·5
Sleep disturbances	No sleep disturbances ICSD-3	849	825·1	88·1
	Sleep disturbances ICSD-3	87	110·9	11·9
Daily screen time	High (≥ 3·5 h/d)	420	475·8	50·8
Following any diet	Yes	179	178	19·0
Physical activity and sedentarity	Active and non-sedentary	62	61·8	6·6
	Active and sedentary	610	578·6	61·8
	Inactive and non-sedentary	38	37·9	4·1
	Inactive and sedentary	226	257	27·5
			Mean	sd
NRF9.3 score (/900)			672·2	96·3

NRF, nutrient-rich foods.

*n* is the number of individuals having this characteristic out of 936.

### Gas-related symptoms and association with socio-demographic and lifestyle characteristics

Table 2 presents the global and the six-symptom dimension IGQ scores in our study population. Mean global IGQ score was 11·9 (sd 11·2) with 55 % of subjects reporting a global score between 0 and 10 and 7 % of subjects reporting no symptoms at all. Among the six dimensions, the highest mean score was found for flatulence with 17·8 (sd 17·4) and the lowest mean score for difficult gas evacuation with 7·3 (sd 15·2). Nearly 30 % of study participants (277/936) reported a high impact of GRS on their QoL with a mean global IGQ score of 23·6 (sd 11·9).

**Table 2.** Distribution of IGQ global score in the study population (*n* 936) (Mean values and standard deviations)

	Mean	sd	Minimum	Maximum	Median	Q1–Q3
Global score	11·9	11·2	0	70·1	8·5	3·9–15·7
Difficult gas evacuation	7·3	15·2	0	100	0	0–12·5
Stomach rumbling	12	17·6	0	100	5	0–17·5
Bad breath	10·8	15·8	0	90	0	0–17·5
Belching	10·5	15·5	0	85	0	0–15
Flatulence	17·8	17·4	0	100	13·3	3·3–26·7
Bloating	12·7	16·6	0	91·7	5·8	0–18·3

IGQ, Intestinal Gas Questionnaire; Q1, first quartile; Q3, third quartile.

Among subjects reporting symptoms, 21 % took an action such as taking drugs, consuming fermented food products (e.g. yogurt), consuming supplements (homeopathy, phytotherapy, probiotics), consulting a physician or starting a specific diet (e.g. low FODMAP).

Using univariate linear regression models, qualitative parameters significantly associated with mean (Q1–Q3) IGQ global score were age (*P* < 0·001), with individuals aged 18–24 years having a higher score (17·7 (6·8–25·3)) *v*. participants aged 65 years old or above (8·1 (1·7–10·7)), daily screen time (*P* < 0·001), with higher screen time associated with a higher score (≥ 3·5 h/d; 13·5 (4·8–19·0)) *v*. lower screen time (< 3·5 h/d; 10·1 (3·5–14·2)), occupation (*P* < 0·001), with unemployed subjects having a higher score (17·0 (6·3–25·4)) *v*. farmer/artisan/trader/business owner (7·6 (1·6–10·5)), education level (*P* < 0·001), with graduate individuals having a higher score (12·26 (2·64–17·86)) *v*. individuals without diploma (4·96 (1·67–8·24)) and smoking (*P* < 0·001), with smokers having a higher score (14·50 (5·56–18·91)) *v*. non-smokers (11·29 (3·55–15·69)). Regarding quantitative parameters, latency to sleep in minutes (*P* = 0·025) and meals taken outside (*P* < 0·001), in front of a screen (*P* < 0·001), with friends (*P* < 0·001) or standing (*P* = 0·005), were all positively correlated with IGQ global score. Results are summarised in Online Resource 1.

### Dietary patterns and association with digestive symptoms

**Fig. 2.**
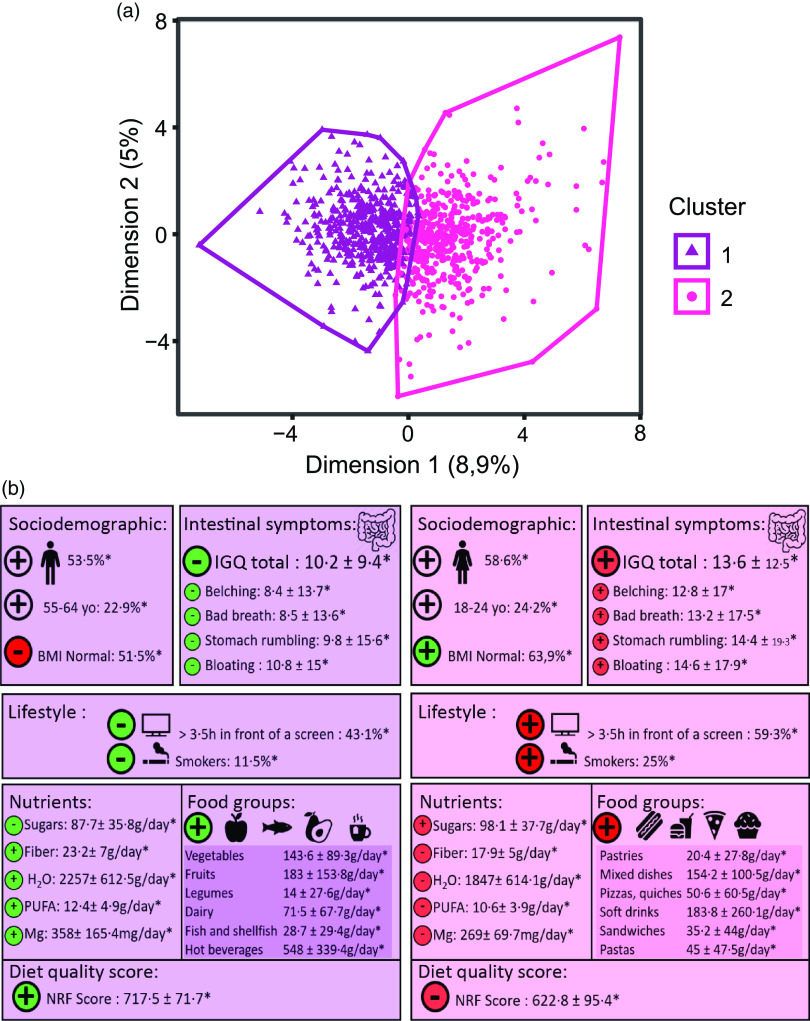
Identification of two dietary patterns and their association with digestive symptoms.

Two different dietary patterns were identified among the study population (Fig. 2 and Online Resource 2). Subjects in cluster 1 (*n* 466), characterised by a lower proportion of women (46·5 %) and smokers, older individuals and declaring less daily screen time, appeared to have healthier dietary patterns with a higher consumption of vegetables, fruits, legumes and dairy products as compared with the total sample average. Subjects in this cluster had overall a higher intake of dietary fibres (23·2 (sd 7) g/d) and a lower intake of sugars (87·7(sd 35·8) g/d). NRF9.3 score in cluster 1 was significantly higher as compared with the total sample average (717·5 (sd 71·7) *v*. 672·2 (sd 96·3); *P* < 0·001). IGQ global score (10·2 (sd 9·4); *P* < 0·001) and belching (8·4 (sd 13·7); *P* < 0·001), bad breath (8·5 (sd 13·6); *P* < 0·001), stomach rumbling (9·8 (sd 15·6); *P* < 0·001) and bloating (10·8 ± 15; *P* < 0·001) dimension scores were lower in cluster 1 as compared with the total sample average.

Subjects in cluster 2 (*n* 470), characterised by a higher proportion of women (58·6 %) and smokers, younger individuals and declaring a higher daily screen time, appeared to have less healthy dietary patterns with a higher consumption of pastries, pizzas and quiches, mixed dishes and soft drinks as compared with the total sample average. Subjects in this cluster had overall a higher intake of sugars (98·1 (sd 37·7) g/d) and a lower intake of dietary fibres (17·9 (sd 5) g/d). NRF9.3 score in cluster 2 was significantly lower as compared with the total sample average (622·8 (sd 95·4) *v*. 672·2 (sd 96·3); *P* < 0·001). IGQ global score (13·6 (sd 12·5); *P* < 0·001) and belching (12·8 (sd 17); *P* < 0·001), bad breath (13·2 (sd 17·5); *P* < 0·001), stomach rumbling (14·4 (sd 19·3); *P* < 0·001) and bloating (14·6 (sd 0·8); *P* < 0·001) dimension scores were higher in cluster 2 as compared with the total sample average.

### Multivariate modelling of associations between symptoms, dietary patterns, socio-demographic and lifestyle characteristics

An age-related decrease in GRS was observed as older participants had a significant reduction of their IGQ global score *v*. participants aged 18–24 years, with, for example, a LS-Means difference of −4·9 points (95 % CI –8·45, −1·31) in participants aged 65 years old or above. Female sex was associated with a significantly higher IGQ global score (+2·7 points (95 % CI 1·19, 4·22)) as compared with males.

Unemployment was associated with a higher IGQ global score (+6·3 points (95 % CI +2·37, +10·15)) *v*. farmer/artisan/trader/business owner (ref.). Regarding lifestyle parameters, in people with regular physical activity, sedentarity was associated with a higher IGQ global score (+3 points (95 % CI +0·14, +5·89)) *v*. non-sedentarity. Moreover, greater daily screen time (≥ 3·5 h/d) and having meals in front of a screen/standing/with family/with friends were significantly associated with a higher IGQ global score (respectively, +2·1 points (95 % CI 0·59, 3·60) *v*. daily screen time < 3·5 h/d, +9·1 points (95 % CI 3·28, 14·93) for having meals standing *v*. seating). Finally, while a greater consumption of pasta (150 g/d; +2·4 points (95 % CI –0·17, 5·02)), fruit juice/nectar (200 ml/d; +1·5 points (95 % CI 0·22, 2·71)) and bread/rusks (50 g/d; +0·8 points (95 % CI 0·21, 1·34)) was associated with a higher IGQ global score, a higher consumption of dairy products (250 g/d; –2·9 points (95 % CI –5·64, –0·24)), water (500 ml/d; –1·0 points (95 % CI –1·84, –0·25)) and fruits (100 g/ d; –0·5 points (95 % CI –1·05, 0·04)) was associated with a lower IGQ global score. Effect size and 95 % CI are summarised in Table 3.

**Table 3.** Factors of association with IGQ global score (multivariate linear model) (95 % confidence intervals)

	Modality	Estimate	95 % CI	Pr( > |t|)
Age				
	18–24 y.o. (ref.)		–	–
	25–34 y.o.	–1·4	–4·65, 1·95	0·4222
	35–44 y.o.	–2·5	–5·80, 0·73	0·1285
	45–54 y.o.	–4·4	–7·58, −1·17	0·0075
	55–64 y.o.	–3·4	–6·80, 0·08	0·0557
	+65 y.o.	–4·9	–8·45, −1·31	0·0074
Physical activity and sedentarity				
	Active and non-sedentary (ref.)		–	–
	Active and sedentary	3·0	0·14, 5·89	0·0398
	Inactive and non-sedentary	–0·5	–4·83, 3·92	0·8370
	Inactive and sedentary	0·0	–3·13, 3·09	0·9903
Sex				
	Male (ref.)			
	Female	2·7	1·19, 4·22	0·0005
Occupation				
	Farmer, artisan, trader. business owner (ref.)			
	Executive, Senior Intellectual Profession, Liberal Profession	3·6	0·46, 6·67	0·0247
	Intermediate occupation	4·8	1·95, 7·71	0·0010
	Employee	3·8	1·05, 6·64	0·0070
	Worker	3·7	−0·09, 7·45	0·0554
	Never employed/inactive	6·3	2·37, 10·15	0·0016
Daily screen time				
	< 3·5 h in front of a screen (ref.)			
	≥ 3·5 h in front of a screen	2·1	0·59, 3·60	0·0063
Meals while standing		9·1	3·28, 14·93	0·0022
Meals with family		4·2	1·17, 7·24	0·0067
Meals in front of a screen		7·2	1·87, 12·52	0·0081
Meals with friends		9·0	2·14, 15·88	0·0102
Food groups (unit)				
	Dairy products (250 g/d)	–2·9	–5·64, −0·24	0·0332
	Water (500 ml/d)	–1·0	–1·84, −0·25	0·0097
	Pastas (150 g/d)	2·4	–0·17, 5·02	0·0665
	Bread and rusks (50 g/d)	0·8	0·21, 1·34	0·0069
	Juices and nectar (200 ml/d)	1·5	0·22, 2·71	0·0209
	Fruits (100 g/d)	–0·5	–1·05, 0·04	0·0669

y.o., years old.

Portion sizes are given based on French standard recommendation; Estimates and 95 % CI of the value factor effects on the IGQ global score were obtained by calculating the LS-Means differences between the value of interest LS-Mean and the reference value LS-Mean within the factor (ref.) for qualitative factors and as a difference of intake equals to the portion size for quantitative factors (e.g. for any other constant factor elsewhere in the model, +65-year-old participants have a significant reduction of −4·9 points (95 % CI –8·45, −1·31) of IGQ global score as compared with 18–24-year-old participants (ref.)).

## Discussion

In this study, we observe for the first time using a validated questionnaire that, with 93 % of subjects reporting at least one gas-related intestinal symptom such as flatulence and bloating in the past 24 h, nearly all adults in the French population experience daily GRS. Furthermore, nearly 30 % of study participants reported a high impact of GRS on their QoL, meaning almost one in three French adults. It is plausible that this group includes some patients diagnosed with a DGBI as 4·2 % of the French adult population is suffering from IBS and 6 % from functional bloating/distension as per Rome IV criteria^(35)^. However, as Rome diagnostic criteria were not used in the present study, this remains to be elucidated.

The rather low average IGQ score found in our study population (mean global score 11·9 (sd 11·2) on a 0–100 scale) can be explained by half of the individuals (51 %) reporting a global score between 0 and 10 and 7 % of subjects reporting no symptoms at all. IGQ scores in our study population were not normally distributed, with most participants below the mean for IGQ global score and all six symptom dimensions. This suggests a large variability as illustrated by median/range values for IGQ global score (8·5 (0–70·1)). Among the six symptom dimensions, the highest score was found for flatulence. This finding is in line with a normal physiological process as on average healthy subjects expel intestinal gas eight times a day^(13,35)^. While difficult gas evacuation, commonly associated with bloating and even abdominal distension, is frequently reported by patients with IBS^(1,36)^, it is the lowest symptom dimension score in our study sample representative of the general population.

Even if IGQ is not a diagnostic tool, there is a consistent gradient of mean IGQ global score between this representative sample of the French general population with 11·9 (sd 11·2) and the scores reported during the IGQ validation study, that is, individuals complaining of several GRS with 33·5 (sd 16·9) and IBS patients with 39·5 (sd 14·7)^(9)^. Indeed, when considering the 30 % of study participants reporting a high impact of GRS on their QoL, their mean IGQ global score reached 23·6 (sd 11·9), thereby representing a continuum between individuals without burden from their symptoms and patients diagnosed with IBS.

In the present study, socio-demographic characteristics such as younger age, female sex or unemployment were associated with a higher IGQ global score. Some of these characteristics (younger age, female sex) are also associated with DGBI like in IBS^(35)^. Available findings from studies conducted in the general population in The Netherlands and USA also report a higher prevalence of GRS in females and a lower prevalence in older individuals (> 65 years old)^(1,3)^. Hence, younger age and female sex both seem to be associated with a higher burden from digestive symptoms.

Regarding lifestyle parameters, greater daily screen time, smoking, having meals in front of a screen/standing/with family/with friends and greater time to fall asleep were all associated with higher severity of symptoms and negative impact on QoL. Greater time to fall asleep has been linked to stress exposure^(37–39)^, and chronic stress and sleeping difficulties have been associated with self-reported IBS and gastrointestinal symptoms in a Swedish population-based study^(40)^. From our knowledge, the other associations found in our study between digestive symptoms and lifestyle parameters had not been reported previously.

We derived two clusters that appear to reflect already known patterns of dietary intake in epidemiological literature. Cluster 1 was associated with a higher consumption of vegetables, legumes, fruits, fish and comprised older individuals, less smokers and surprisingly more abnormal BMI.

These lifestyle characteristics (less smokers, more overweight and older individuals) were also found in another study exploring French dietary patterns, which described them as ‘Prudent pattern’.^(41)^. These unexpected findings may indicate that individuals with overweight or obesity may have changed or improved their diet because of their medical conditions, or it may be that while the overall quality of their diet is good, portion sizes are too high.

Conversely, cluster 2 reflected a higher consumption of mixed dishes, pastries, pizzas, quiches, sandwiches and comprised younger participants with a higher proportion of individuals smoking and living in an urban area. Other studies have found the same association between this dietary pattern characterised by a higher consumption of ‘convenient food’ and this kind of socio-demographic characteristics^(41–43)^.

Individuals with lower diet nutritional quality, that is, a dietary pattern higher in foods ‘on the go’, higher in sugars and lower in dietary fibres, reported more severe symptoms and a higher impact on their QoL as compared with the total sample average. Conversely, higher diet nutritional quality, that is, dietary patterns lower in sugars and higher in dietary fibres intake with more unprocessed foods, was associated with less burden from GRS. Our results are consistent with previous studies reporting that an increased proportion of ultra-processed foods in the diet was associated with a higher prevalence of IBS^(44,45)^.

In our study, increased daily consumption of dairy products and water was associated with less severe symptoms and impact on QoL. These beneficial associations are driven by consumption of additional daily portions of 500 ml of water and 250 g of dairy products (e.g. 2 pots of 125 g yogurt). This dairy consumption is in adequacy with the French National guidelines (Plan National Nutrition Santé) which recommend consuming two dairy products per day^(46)^. Regarding water intake, our results are again in accordance with the guidelines, this time at the European level from the EFSA^(47)^ which recommends about 2·5 l of water per day for men including 2 l from beverages and 2 l for women including 1·6 l from beverages.

Strengths of our study include a quota-based sampling representative of the French adult population (considering age, socio-economic status, geographical region, town size and household size) that allows extrapolation of findings from the present cohort to the general population.

Another strength is the robustness of findings from multivariate analysis as significantly associated factors to IGQ global scores were consistent when using different models, that is, both backward and forward methods. This allows to affirm with reliability the associations revealed in this study such as younger age, female sex, unemployment or greater daily screen time, all associated with higher IGQ scores, and higher consumption of dairy products, water and fruits, all associated with lower IGQ scores.

Finally, the detailed dietary assessment method (7-d e-food diary) and the thorough characterisation of individuals through numerous socio-demographic and lifestyle parameters could help designing future dietary and lifestyle interventions aimed at reducing the burden of GRS in the French adult population.

Limitations from our study include results that are based on exploratory associations, not causality, therefore preventing us from identifying the factors eliciting GRS. As this is the first use of IGQ in combination with socio-demographic, lifestyle and dietary parameters in a nationally representative sample, no *a priori* hypothesis was made regarding the prevalence of GRS in French adults, and we did not *a priori* consider the power of the study. All conclusions discussed in this article are therefore considered explorative.

Another limitation is the over-simplification of dietary habits in the general population with two clusters, which is not representative of the real-life diversity in dietary habits.

Then, as it is the first use of the IGQ following its recent validation, the threshold used to define individuals reporting a high impact of GRS on their QoL was chosen arbitrarily. This threshold should thus be validated in other cohorts.

Finally, the proportion of individuals suffering from GRS and potentially diagnosed with a DGBI such as IBS or functional bloating/distension could not be determined as Rome diagnostic criteria were not part of this survey.

In conclusion, this is the first study assessing the prevalence of GRS in the general population with a validated questionnaire. We showed that GRS are associated with a lower QoL and affect almost all adults daily in the French population, with flatulence being the most frequently reported symptom. Nearly 30 % of study participants reported a high impact of GRS on their QoL. Regarding socio-demographic factors associated with GRS, we confirm here previous findings showing that younger age and female sex seem to be robustly associated with a higher burden from digestive symptoms, and we report novel associations with employment status. Regarding lifestyle parameters associated with GRS, we show that previously reported associations with sleeping difficulties are also found in our sample from the general population. We also report novel associations with daily screen time, smoking status and context of meals. Finally, we report for the first time that GRS are associated with diet quality and dietary patterns in a sample of the French adult population.

This previously underestimated burden of GRS in the general population means that solutions are either lacking or ineffective. Awareness from lay audience, medical professionals (gastroenterologists, general practitioners, dietitians) and overall scientific community regarding this public health issue needs to be developed. In terms of future research, it would be interesting to extend the present findings to other geographies, including non-European countries, and to confirm by dietary and lifestyle interventions that GRS prevalence and impact can be reduced using IGQ as endpoint.

## Supplementary material

For supplementary material accompanying this paper visit https://doi.org/10.1017/S0007114522001593. click here to view supplementary material

## References

[1] Jiang X , Locke GR , Choung RS , et al. (2008) Prevalence and risk factors for abdominal bloating and visible distention: a population-based study. Gut 57, 756–763. 18477677 10.1136/gut.2007.142810 PMC2581929

[2] Tuteja AK , Talley NJ , Joos SK , et al. (2008) Abdominal bloating in employed adults: prevalence, risk factors, and association with other bowel disorders. Am J Gastroenterol 103, 1241–1248. 18422817 10.1111/j.1572-0241.2007.01755.x

[3] Tielemans MM , Jaspers Focks J , van Rossum LG , et al. (2013) Gastrointestinal symptoms are still prevalent and negatively impact health-related quality of life: a large cross-sectional population based study in The Netherlands. PLOS ONE 8, e69876. 23922836 10.1371/journal.pone.0069876 PMC3726702

[4] L’Heureux-Bouron D , Legrain-Raspaud S , Carruthers HR , et al. (2018) Minor digestive symptoms and their impact in the general population: a cluster analysis approach. Therap Adv Gastroenterol 11, 1756284818768814. 10.1177/1756284818768814 PMC5946602 29760783

[5] Vivier H , Ross EJ & Cassisi JE (2020) Classification of gastrointestinal symptom patterns in young adults. BMC Gastroenterol 20, 326. 33023502 10.1186/s12876-020-01478-7 PMC7542131

[6] Gralnek IM , Hays RD , Kilbourne A , et al. (2000) The impact of irritable bowel syndrome on health-related quality of life. Gastroenterology 119, 654–660. 10982758 10.1053/gast.2000.16484

[7] El-Serag HB (2003) Impact of irritable bowel syndrome: prevalence and effect on health-related quality of life. Rev Gastroenterol Disord 3, S3–11. 12775997

[8] Kanazawa M , Miwa H , Nakagawa A , et al. (2016) Abdominal bloating is the most bothersome symptom in irritable bowel syndrome with constipation (IBS-C): a large population-based Internet survey in Japan. Biopsychosoc Med 10, 19. 27274765 10.1186/s13030-016-0070-8 PMC4893246

[9] Duracinsky M , Archbold S , Lobo B , et al. (2021) The Intestinal Gas Questionnaire (IGQ): psychometric validation of a new instrument for measuring gas-related symptoms and their impact on daily life among general population and irritable bowel syndrome. Neurogastroenterol Motil 34, e14202. 10.1111/nmo.14202 PMC9285021 34145682

[10] Bohn L , Storsrud S , Tornblom H , et al. (2013) Self-reported food-related gastrointestinal symptoms in IBS are common and associated with more severe symptoms and reduced quality of life. Am J Gastroenterol 108, 634–641. 23644955 10.1038/ajg.2013.105

[11] Monsbakken KW , Vandvik PO & Farup PG (2006) Perceived food intolerance in subjects with irritable bowel syndrome-- etiology, prevalence and consequences. Eur J Clin Nutr 60, 667–672. 16391571 10.1038/sj.ejcn.1602367

[12] Skodje GI , Sarna VK , Minelle IH , et al. (2018) Fructan, rather than gluten, induces symptoms in patients with self-reported non-celiac gluten sensitivity. Gastroenterology 154, 529–539.e522. 29102613 10.1053/j.gastro.2017.10.040

[13] Manichanh C , Eck A , Varela E , et al. (2014) Anal gas evacuation and colonic microbiota in patients with flatulence: effect of diet. Gut 63, 401–408. 23766444 10.1136/gutjnl-2012-303013 PMC3933177

[14] Linlawan S , Patcharatrakul T , Somlaw N , et al. (2019) Effect of rice, wheat, and mung bean ingestion on intestinal gas production and postprandial gastrointestinal symptoms in non-constipation irritable bowel syndrome patients. Nutrients 11, 2061. 31484315 10.3390/nu11092061 PMC6771122

[15] El-Salhy M , Ystad SO , Mazzawi T , et al. (2017) Dietary fiber in irritable bowel syndrome (review). Int J Mol Med 40, 607–613. 28731144 10.3892/ijmm.2017.3072 PMC5548066

[16] Halmos EP , Power VA , Shepherd SJ , et al. (2014) A diet low in FODMAPs reduces symptoms of irritable bowel syndrome. Gastroenterology 146, 67–75.e65. 24076059 10.1053/j.gastro.2013.09.046

[17] O’Keeffe M , Jansen C , Martin L , et al. (2018) Long-term impact of the low-FODMAP diet on gastrointestinal symptoms, dietary intake, patient acceptability, and healthcare utilization in irritable bowel syndrome. Neurogastroenterol Motil 30, e13154. 10.1111/nmo.13154 28707437

[18] Staudacher HM , Whelan K , Irving PM , et al. (2011) Comparison of symptom response following advice for a diet low in fermentable carbohydrates (FODMAPs) versus standard dietary advice in patients with irritable bowel syndrome. J Hum Nutr Diet 24, 487–495. 21615553 10.1111/j.1365-277X.2011.01162.x

[19] Schafer F , Faviez C , Voillot P , et al. (2020) Mapping and modeling of discussions related to gastrointestinal discomfort in French-speaking online forums: results of a 15-year retrospective infodemiology study. J Med Internet Res 22, e17247. 33141087 10.2196/17247 PMC7671840

[20] Kant AK (2004) Dietary patterns and health outcomes. J Am Diet Assoc 104, 615–635. 15054348 10.1016/j.jada.2004.01.010

[21] Hu FB (2002) Dietary pattern analysis: a new direction in nutritional epidemiology. Curr Opin Lipidol 13, 3–9. 11790957 10.1097/00041433-200202000-00002

[22] Drewnowski A (2009) Defining nutrient density: development and validation of the nutrient rich foods index. J Am Coll Nutr 28, 421S–426S. 20368382 10.1080/07315724.2009.10718106

[23] Kennedy ET , Ohls J , Carlson S , et al. (1995) The Healthy Eating Index: design and applications. J Am Diet Assoc 95, 1103–1108. 7560680 10.1016/S0002-8223(95)00300-2

[24] Verger EO , Mariotti F , Holmes BA , et al. (2012) Evaluation of a diet quality index based on the probability of adequate nutrient intake (PANDiet) using national French and US dietary surveys. PLoS One 7, e42155. 22870293 10.1371/journal.pone.0042155 PMC3411671

[25] Kristeller JL & Rodin J (1989) Identifying eating patterns in male and female undergraduates using cluster analysis. Addict Behav 14, 631–642. 2618846 10.1016/0306-4603(89)90005-1

[26] Bellisle F , Hebel P , Colin J , et al. (2014) Consumption of whole grains in French children, adolescents and adults. Br J Nutr 112, 1674–1684. 25300424 10.1017/S0007114514002670 PMC4234471

[27] Francou A , Hebel P , Braesco V , et al. (2015) Consumption patterns of fruit and vegetable juices and dietary nutrient density among french children and adults. Nutrients 7, 6073–6087. 26213964 10.3390/nu7085268 PMC4555108

[28] Keys A , Fidanza F , Karvonen MJ , et al. (1972) Indices of relative weight and obesity. J Chronic Dis 25, 329–343. 4650929 10.1016/0021-9681(72)90027-6

[29] ANSES (2017) ANSES OPINION and REPORT on the update of the food consumption database and the estimation of the nutritional intakes of individuals living in France through the implementation of the 3rd national individual study of food consumption (INCA3 study). France: ANSES.

[30] Drewnowski A (2010) The Nutrient Rich Foods Index helps to identify healthy, affordable foods. Am J Clin Nutr 91, 1095S–1101S. 20181811 10.3945/ajcn.2010.28450D

[31] Chassany O , Tugaut B , Marrel A , et al. (2015) The Intestinal Gas Questionnaire: development of a new instrument for measuring gas-related symptoms and their impact on daily life. Neurogastroenterol Motil 27, 885–898. 25846412 10.1111/nmo.12565

[32] Schofield WN (1985) Predicting basal metabolic rate, new standards and review of previous work. Hum Nutr Clin Nutr 39, 5–41. 4044297

[33] Black AE (2000) Critical evaluation of energy intake using the Goldberg cut-off for energy intake:basal metabolic rate. A practical guide to its calculation, use and limitations. Int J Obes Relat Metab Disord 24, 1119–1130. 11033980 10.1038/sj.ijo.0801376

[34] Akaike H (1974) A new look at the statistical model identification. IEEE Trans Autom Control 19, 716–723.

[35] Sperber AD , Bangdiwala SI , Drossman DA , et al. (2021) Worldwide prevalence and burden of functional gastrointestinal disorders, results of Rome foundation global study. Gastroenterology 160, 99–114.e113. 32294476 10.1053/j.gastro.2020.04.014

[36] Chang L , Lee OY , Naliboff B , et al. (2001) Sensation of bloating and visible abdominal distension in patients with irritable bowel syndrome. Am J Gastroenterol 96, 3341–3347. 11774947 10.1111/j.1572-0241.2001.05336.x

[37] Buckley TM & Schatzberg AF (2005) On the interactions of the hypothalamic-pituitary-adrenal (HPA) axis and sleep: normal HPA axis activity and circadian rhythm, exemplary sleep disorders. J Clin Endocrinol Metab 90, 3106–3114. 15728214 10.1210/jc.2004-1056

[38] Basta M , Chrousos GP , Vela-Bueno A , et al. (2007) Chronic insomnia and stress system. Sleep Med Clin 2, 279–291. 18071579 10.1016/j.jsmc.2007.04.002 PMC2128619

[39] Kalmbach DA , Anderson JR & Drake CL (2018) The impact of stress on sleep: pathogenic sleep reactivity as a vulnerability to insomnia and circadian disorders. J Sleep Res 27, e12710. 29797753 10.1111/jsr.12710 PMC7045300

[40] Zejnelagic J & Ohlsson B (2021) Chronic stress and poor sleeping habits are associated with self-reported IBS and poor psychological well-being in the general population. BMC Res Notes 14, 280. 34294119 10.1186/s13104-021-05688-4 PMC8296624

[41] Kesse-Guyot E , Bertrais S , Peneau S , et al. (2009) Dietary patterns and their sociodemographic and behavioural correlates in French middle-aged adults from the SU.VI.MAX cohort. Eur J Clin Nutr 63, 521–528. 18212801 10.1038/sj.ejcn.1602978

[42] Naska A , Fouskakis D , Oikonomou E , et al. (2006) Dietary patterns and their socio-demographic determinants in 10 European countries: data from the DAFNE databank. Eur J Clin Nutr 60, 181–190. 16278696 10.1038/sj.ejcn.1602284

[43] Torres MJ , Sabate JM , Bouchoucha M , et al. (2018) Food consumption and dietary intakes in 36,448 adults and their association with irritable bowel syndrome: Nutrinet-Sante study. Therap Adv Gastroenterol 11, 1756283X17746625. 10.1177/1756283X17746625 PMC5788087 29399039

[44] Buscail C , Sabate JM , Bouchoucha M , et al. (2017) Western dietary pattern is associated with irritable bowel syndrome in the French NutriNet cohort. Nutrients 9, 986. 28880222 10.3390/nu9090986 PMC5622746

[45] Schnabel L , Buscail C , Sabate JM , et al. (2018) Association between ultra-processed food consumption and functional gastrointestinal disorders: results From the French NutriNet-Sante cohort. Am J Gastroenterol 113, 1217–1228. 29904158 10.1038/s41395-018-0137-1

[46] Santé-Publique-France (2001) Programme National Nutrition Santé (PNNS) – Manger Bouger. http://www.mangerbouger.fr/PNNS (accessed March 2022).

[47] Additives EPoF (2010) Scientific opinion on dietary reference values for water. EFSA J 8, 1459.

