# Supplementary material for: A poor diet quality is associated with more gas-related symptoms and a decreased quality of life in French adults
Source: Br J Nutr. 2022 May 23;129(4):715–24. doi: 10.1017/S0007114522001593 (PMC9899566; doi:10.1017/S0007114522001593)
Supplement: Supplementary file 1 [file S0007114522001593sup.zip › S0007114522001593sup001.docx]

| Online ressource1. Univariate factors of association with IGQ Global Score | | | | |
| --- | --- | --- | --- | --- |
|  | **Population** | | **IGQ Global Score** | ***p-V*alue** |
|  | n (%) | Weighted n (%) | mean [Q1 - Q3] |  |
|  |  |  |  |  |
| IGQ Global Score | 936 | 936 | 11.86 [3.89-15.69] |  |
| **Physical activity and sedentarity** |  |  |  | **<0.001** |
| Active and non-sedentary | 62 (6.6%) | 61.8 (6.6) | 7.98 [1.94-10.94] |  |
| Active and sedentary | 610 (65.2%) | 578.6 (61.8) | 13.16 [4.58-17.87] |  |
| Inactive and non-sedentary | 38 (4.1%) | 37.9 (4.1) | 9.97 [5.56-15.87] |  |
| Inactive and sedentary | 226 (24.1%) | 257.7 (27.5) | 10.13 [3.06-14.17] |  |
| **Age group (years)** |  |  |  | **<0.001** |
| 18-24 | 99 (10.6%) | 116.2 (12.4) | 17.72 [6.78-25.30] |  |
| 25-34 | 181 (19.3%) | 119.7 (12.8) | 14.28 [7.6-18.48] |  |
| 35-44 | 125 (13.4%) | 140.2 (15.0) | 13.47 [5.69-19.09] |  |
| 45-54 | 180 (19.2%) | 177.2 (18.9) | 10.72 [2.79-14.82] |  |
| 55-64 | 194 (20.7%) | 149.7 (16.0) | 11.10 [3.89-14.31] |  |
| 65+ | 157 (16.8%) | 233.1 (24.9) | 8.07 [1.67-10.69] |  |
| **Gas-related symptoms** |  |  |  | **<0.001** |
| Individuals mostly affected by their symptoms | 277 (29.6%) | 280.1 (29.9) | 23.61 [14.44-31.39] |  |
| Individuals least affected by their symptoms | 659 (70.4%) | 656.9 (70.2) | 6.84 [2.74-10.28] |  |
| **Occupation** |  |  |  |  |
| Farmer, artisan, trader, business owner | 29 (3.1%) | 75.9 (8.1) | 7.65 [1.59-10.46] | **<0.001** |
| Executive, Senior Intellectual, Liberal | 358 (38.2%) | 114.2 (12.2) | 9.99 [2.9-14.57] |  |
| Employee | 248 (26.5%) | 190.9 (20.4) | 11.96 [4.58-15.69] |  |
| Worker | 38 (4.1%) | 371.5 (39.7) | 12.65 [3.75-18.06] |  |
| Intermediate occupation | 167 (17.8%) | 60.1 (6.4) | 10.85 [3.47-14.72] |  |
| Never employed / Inactive | 96 (10.3%) | 123.4 (13.2) | 17.03 [6.33-25.39] |  |
| **Education** |  |  |  |  |
| No diploma | 16 (1.7%) | 24.9 (2.7) | 4.96 [1.67-8.24] | **<0.001** |
| Lower than High School Degree | 60 (6.4%) | 95.9 (10.2) | 10.32 [6.11-13.35] |  |
| High School | 228 (24.4%) | 321.0 (34.3) | 12.33 [3.75-15.98] |  |
| Undergraduate | 387 (41.3%) | 380.6 (40.7) | 12.16 [4.44-16.67] |  |
| Graduate | 234 (25%) | 104.0 (11.1) | 12.26 [2.64-17.86] |  |
| **Smoking** |  |  |  | **<0.001** |
| Yes | 165 (17.6%) | 168.2 (18.0) | 14.50 [5.56-18.91] |  |
| No | 763 (81.5%) | 759.3 (81.1) | 11.29 [3.55-15.69] |  |
| **BMI** |  |  |  | 0.359 |
| Underweight | 34 (3.6%) | 31.3 (3.3) | 14.68 [5.56-18.13] |  |
| Normal | 597 (63.8%) | 538. (57.5) | 11.26 [3.47-15.42] |  |
| Overweight | 230 (24.6%) | 277.7 (29.7) | 12.17 [4.17-15.94] |  |
| Obese | 75 (8%) | 89.0 (9.5) | 13.51 [4.86-21.67] |  |
| **Diet without gluten** |  |  |  | 0.37 |
| Yes | 26 (2.8%) | 25.2 (2.7) | 14.53 [5.22-16.29] |  |
| No | 910 (97.2%) | 910.8 (97.3) | 11.78 [3.89-15.69] |  |
| **Diet without lactose** |  |  |  | 0.818 |
| Yes | 47 (5%) | 39.3 (4.2) | 11.94 [3.62-16.53] |  |
| No | 889 (95%) | 896.7 (95.8) | 11.85 [3.89-15.69] |  |
| **Following a weight-loss diet** |  |  |  | 0.698 |
| Yes | 179 (19.1%) | 178.0 (19.0) | 12.48 [3.68-17.86] |  |
| No | 757 (80.9%) | 758.0 (81.0) | 11.71 [4.03-15.69] |  |
| **Income** |  |  |  | 0.226 |
| Less than 1,700 € / 1,967 USD | 136 (14.5%) | 172.2 (18.4) | 13.69 [5.08-16.67] |  |
| From 1,700€/1,967 USD to 3,000 €/3,471 USD | 206 (22%) | 217.2 (23.2) | 12.44 [3.63-16.16] |  |
| From 3,000€/3,471 USD to 4,500 €/5,206 USD | 230 (24.6%) | 228.8 (24.4) | 10.32 [3.47-15.14] |  |
| More than 4,500 €/5,206 USD | 141 (15.1%) | 93.6 (10.0) | 11.73 [3.19-16.57] |  |
| **Sex** |  |  |  | 0.164 |
| Male | 364 (38.9%) | 446.5 (47.7) | 10.60 [4.17-14.31] |  |
| Female | 572 (61.1%) | 489.5 (52.3) | 13.00 [3.75-17.97] |  |
| **Sleep disturbances** |  |  |  | 0.366 |
| No sleep disturbances ICSD-3 | 849 (90.7%) | 825.1 (88.2) | 11.63 [3.89-15.56] |  |
| Sleep disturbances ICSD-3 | 87 (9.3%) | 110.9 (11.8) | 13.53 [4.31-20.28] |  |
| **Daily screen time** |  |  |  | **<0.001** |
| Low (< 3.5 hours) | 516 (55.1%) | 460.2 (49.2) | 10.10 [3.47-14.17] |  |
| High (≥ 3.5 hours) | 420 (44.9%) | 475.8 (50.8) | 13.55 [4.78-19.03] |  |
| **Living area** |  |  |  | 0.875 |
| Rural | 190 (20.3%) | 193.8 (20.7) | 11.01 [3.06-14.18] |  |
| 2 to 20,000 inhabitants | 156 (16.7%) | 189.1 (20.2) | 11.62 [4.86-15.69] |  |
| 20 to 100,000 inhabitants | 121 (12.9%) | 126.4 (13.5) | 11.13 [4.31-14.17] |  |
| >100,000 inhabitants | 313 (33.4%) | 276.1 (29.5) | 12.15 [3.06-15.97] |  |
| Paris | 156 (16.7%) | 150.7 (16.1) | 13.31 [5.14-18.47] |  |
| **Vegetarian** |  |  |  | 0.283 |
| Yes | 24 (2.6%) | 23.3 (2.5) | 14.58 [6.71-18.19] |  |
| No | 912 (97.4%) | 912.7 (97.5) | 11.79 [3.89-15.69] |  |
| **Type of household** |  |  |  | 0.701 |
| Single | 210 (22.4%) | 191.9 (20.5) | 11.34 [4.17-16.43] |  |
| Couple without children | 302 (32.3%) | 379.1 (40.5) | 11.23 [4.17-14.44] |  |
| Couple with child(ren) | 337 (36%) | 254.6 (27.2) | 12.07 [3.67-17.24] |  |
| Single parent family | 40 (4.3%) | 84.3 (9.0) | 15.88 [4.34-29.22] |  |
| Other | 47 (5%) | 26.2 (2.8) | 9.85 [3.77-17.94] |  |
| **QUANTITATIVE PARAMETERS (unit)** |  |  | **Linear regression coefficient** | ***p-V*alue** |
| Height (cm) |  |  | -0.1398 | **0.001** |
| Weight (kg) |  |  | -0.0239 | 0.331 |
| **Food items** |  |  |  |  |
| Alcoholic beverages (ml/day) |  |  | -0.0041 | 0.064 |
| Hot beverages (ml/day) |  |  | -0.0029 | **0.01** |
| Breakfast cereals (g/day) |  |  | -0.0122 | 0.544 |
| Water intake (ml/day) |  |  | -0.0010 | 0.195 |
| Cheese (g/day) |  |  | -0.0229 | **0.044** |
| Fruits (g/day) |  |  | -0.0104 | **<0.001** |
| Dry fruits (g/day) |  |  | 0.0275 | 0.797 |
| Seeds (g/day) |  |  | -0.0184 | 0.711 |
| Juices and nectars (ml/day) |  |  | 0.0058 | **0.067** |
| Milk (ml/day) |  |  | 0.0090 | 0.07 |
| Vegetables (g/day) |  |  | -0.0096 | **0.025** |
| Dry vegetables (g/day) |  |  | 0.0007 | 0.965 |
| Bread and rusks (g/day) |  |  | -0.0113 | **0.032** |
| Pasta (g/day) |  |  | 0.0329 | **<0.001** |
| Rice and semolina (g/day) |  |  | 0.0065 | 0.435 |
| Soup (g/day) |  |  | -0.0126 | **<0.001** |
| Dairy products (g/day) |  |  | -0.0196 | **<0.001** |
| Energy (kcal/day) |  |  | 0.0004 | 0.596 |
| Water (g/day) |  |  | -0.0016 | **0.004** |
| Polyols (g/day) |  |  | 0.0788 | 0.759 |
| Fibre (g/day) |  |  | -0.1027 | 0.061 |
| Alcohol (g/day) |  |  | -0.0543 | **0.02** |
| Magnesium (mg/day) |  |  | -0.0074 | **0.005** |
| **Lifestyle** |  |  |  |  |
| Latency to sleep (min) |  |  | 0.0465 | **0.025** |
| Meals taken at home (%) |  |  | -8.7557 | **<0.001** |
| Meals taken outside (%) |  |  | 8.7557 | **<0.001** |
| Meals taken alone (%) |  |  | -1.1297 | 0.449 |
| Meals taken with family (%) |  |  | -2.8019 | **0.036** |
| Meals taken with friends (%) |  |  | 14.5326 | **<0.001** |
| Meal taken with others (%) |  |  | 8.8447 | **0.01** |
| Meals taken in front of TV (%) |  |  | -0.4971 | 0.746 |
| Meals taken in front of screen (%) |  |  | 12.7356 | **<0.001** |
| Meals taken in front of something else (%) |  |  | -6.2870 | **0.005** |
| Meals taken in front of nothing (%) |  |  | -0.9950 | 0.493 |
| Meals taken standing (%) |  |  | 7.8901 | **0.005** |
| Meals taken seated (%) |  |  | -7.8901 | **0.005** |
| ***Footnote:*** *For qualitative parameters the Kruskall-Wallis test was used for comparing means of the IGQ global score within modalities for parameters with more than two levels, and the Wilcoxon-Mann-Whitney test was used for binary parameters. For quantitative parameters, linear regression models were used to assess the relationship with IGQ global score and factors of interest, the Student T-test was used to test the nullity linear regression coefficient.* | | | | |
