# Supplementary material for: A poor diet quality is associated with more gas-related symptoms and a decreased quality of life in French adults
Source: Br J Nutr. 2022 May 23;129(4):715–24. doi: 10.1017/S0007114522001593 (PMC9899566; doi:10.1017/S0007114522001593)
Supplement: Supplementary file 1 [file S0007114522001593sup.zip › S0007114522001593sup002.docx]

| Online resource 2: Subject’s characteristics in the two dietary clusters | | | | | |
| --- | --- | --- | --- | --- | --- |
|  | **Cluster 2** | ***p-V*alue** | **Cluster 1** | ***p-V*alue** | **Total population** |
|  | (n=470) |  | (n=466) |  | (n=936) |
|  | n (%) |  | n (%) |  | n (%) |
| **Physical activity and sedentarity** |  |  |  |  |  |
| Active and non-sedentary | 23 (4.8) | 0.044 | 38 (8.2) | 0.044 | 61 (6.5) |
| Active and sedentary | 320 (68.2) | <0.001 | 261 (56.0) | <0.001 | 581(62.0) |
| Inactive and non-sedentary | 13 (2.8) | 0.041 | 24 (5.2) | 0.088 | 37 (4.0) |
| Inactive and sedentary | 114 (24.2) | 0.023 | 143 (30.6) | 0.034 | 257 (27.5) |
| **Age group (years)** |  |  |  |  |  |
| 18-24 | 114 (24.2) | <0.001 | 7 (1.6) | <0.001 | 121 (12.9) |
| 25-34 | 93 (19.7) | <0.001 | 30 (6.4) | <0.001 | 123 (13.1) |
| 35-44 | 107 (22.8) | <0.001 | 36 (7.8) | <0.001 | 143 (15.3) |
| 45-54 | 88 (18.7) | 0.905 | 89 (19.1) | 0.905 | 177 (18.9) |
| 55-64 | 39 (8.4) | <0.001 | 107 (22.9) | <0.001 | 146 (15.6) |
| 65+ | 29 (6.2) | <0.001 | 197 (42.2) | <0.001 | 226 (24.2) |
| **Individuals mostly affected by their symptoms** | 158 (33.7) | 0.015 | 123 (26.5) | 0.015 | 281 (30.0) |
| **Occupation** |  |  |  |  |  |
| Farmer, artisan, trader, business owner | 22 (4.7) | <0.001 | 53 (11.3) | <0.001 | 75 (8.0) |
| Executive, Senior Intellectual, Liberal | 38 (8.0) | <0.001 | 74 (16.0) | <0.001 | 112 (12.0) |
| Employee | 208 (44.3) | 0.006 | 165 (35.4) | 0.006 | 373 (39.8) |
| Worker | 29 (6.2) | 0.851 | 31 (6.6) | 0.851 | 60 (6.4) |
| Intermediate occupation | 66 (14.1) | <0.001 | 122 (26.2) | <0.001 | 188 (20.1) |
| Never employed / Inactive | 107 (22.7) | <0.001 | 21 (4.5) | <0.001 | 128 (13.7) |
| **Education** |  |  |  |  |  |
| No diploma | 10 (2.2) | 0.436 | 14 (3.1) | 0.436 | 24 (2.6) |
| Lower than High School Degree | 34 (7.2) | 0.003 | 61 (13.0) | 0.003 | 95 (10.1) |
| High School | 169 (36.0) | 0.312 | 153 (32.7) | 0.312 | 322 (34.4) |
| Undergraduate | 192 (40.8) | 0.932 | 189 (40.6) | 0.932 | 381 (40.7) |
| Graduate | 59 (12.6) | 0.135 | 45 (9.7) | 0.198 | 104 (11.1) |
| Missing | 6 (1.2) | 0.660 | 4 (0.9) | 0.660 | 10 (1.1) |
| **Smoking** |  |  |  |  |  |
| Yes | 117 (25.0) | <0.001 | 54 (11.5) | <0.001 | 171 (18.3) |
| No | 347 (73.8) | <0.001 | 409 (87.8) | <0.001 | 756 (80.7) |
| Missing | 6 (1.2) | 0.66 | 3 (0.7) | 0.282 | 9 (1.0) |
| **BMI** |  |  |  |  |  |
| Underweight | 24 (5.2) | 0.003 | 8 (1.7) | 0.003 | 32 (3.4) |
| Normal | 300 (63.9) | <0.001 | 240 (51.5) | <0.001 | 540 (57.7) |
| Overweight | 124 (26.3) | 0.031 | 153 (32.8) | 0.031 | 277 (29.6) |
| Obese | 22 (4.6) | <0.001 | 65 (14.0) | <0.001 | 87 (9.3) |
| **Diet without gluten** |  |  |  |  |  |
| Yes | 8 (1.7) | 0.112 | 17 (3.6) | 0.045 | 25 (2.7) |
| No | 462 (98.3) | 0.112 | 449 (96.4) | 0.112 | 911 (97.3) |
| **Diet without lactose** |  |  |  |  |  |
| Yes | 12 (2.6) | 0.029 | 26 (5.6) | 0.012 | 38 (4.1) |
| No | 458 (97.4) | 0.029 | 440 (94.4) | 0.029 | 898 (95.9) |
| **Following a weight-loss diet** |  |  |  |  |  |
| Yes | 98 (20.9) | 0.144 | 81 (17.3) | 0.144 | 179 (19.1) |
| No | 372 (79.1) | 0.144 | 385 (82.7) | 0.144 | 757 (80.9) |
| **Incomes** |  |  |  |  |  |
| Less than 1,700 € | 105 (22.3) | 0.003 | 69 (14.9) | 0.005 | 174 (18.6) |
| From 1,700 € to 3,000 € | 105 (22.4) | 0.551 | 111 (23.9) | 0.551 | 216 (23.1) |
| From 3,000 € to 4,500 € | 93 (19.8) | 0.001 | 134 (28.7) | 0.002 | 227 (24.3) |
| More than 4,500 € | 29 (6.2) | <0.001 | 63 (13.5) | <0.001 | 92 (9.8) |
| Refusal to disclose | 138 (29.3) | <0.001 | 89 (19.0) | <0.001 | 227 (24.2) |
| **Sex** |  |  |  |  |  |
| Female | 276 (58.6) | <0.001 | 217 (46.5) | <0.001 | 493 (52.7) |
| Male | 194 (41.4) | <0.001 | 249 (53.5) | <0.001 | 443 (47.3) |
| **Falling asleep** |  |  |  |  |  |
| No sleep disturbances ICSD-3 | 405 (86.1) | 0.047 | 420 (90.1) | 0.047 | 825 (88.1) |
| Sleep disturbances ICSD-3 | 65 (13.9) | 0.074 | 46 (9.9) | 0.074 | 111 (11.9) |
| **Daily screen time** |  |  |  |  |  |
| Low (< 3.5 hours) | 191 (40.7) | <0.001 | 265 (56.9) | <0.001 | 456 (48.7) |
| High (≥ 3.5 hours) | 279 (59.3) | <0.001 | 201 (43.1) | <0.001 | 480 (51.3) |
| **Living area** |  |  |  |  |  |
| Rural | 94 (20.0) | 0.646 | 99 (21.3) | 0.646 | 193 (20.6) |
| 2 to 20,000 inhabitants | 80 (17.0) | 0.018 | 108 (23.2) | 0.018 | 188 (20.1) |
| 20 to 100,000 inhabitants | 59 (12.6) | 0.528 | 67 (14.3) | 0.411 | 126 (13.5) |
| >100,000 inhabitants | 145 (30.8) | 0.399 | 131 (28.2) | 0.399 | 276 (29.5) |
| Paris | 92 (19.6) | 0.009 | 61 (13.0) | 0.005 | 153 (16.3) |
| **Vegetarian** |  |  |  |  |  |
| Yes | 15 (3.2) | 0.216 | 9 (1.8) | 0.216 | 24 (2.6) |
| No | 455 (96.8) | 0.098 | 457 (98.2) | 0.216 | 912 (97.4) |
| **Type of household** |  |  |  |  |  |
| Single | 99 (21.1) | 0.616 | 93 (20.0) | 0.616 | 192 (20.5) |
| Couple without children | 124 (26.3) | <0.001 | 249 (53.5) | <0.001 | 373 (39.8) |
| Couple with child(ren) | 163 (34.7) | <0.001 | 95 (20.4) | <0.001 | 258 (27.6) |
| Single parent family | 63 (13.5) | <0.001 | 23 (4.8) | <0.001 | 86 (9.2) |
| Other | 21 (4.4) | 0.003 | 6 (1.3) | 0.010 | 27 (2.9) |
|  | **Mean (SD)** | **p-value** | **Mean (SD)** | **p-value** | **Mean (SD)** |
| Time to fall asleep (min) | 20.1 (18.5) | <0.001 | 15.7 (16.4) | <0.001 | 17.8 (17.6) |
| Weight (kg) | 68.1 (14.2) | <0.001 | 73.6 (14.8) | <0.001 | 70.9 (14.8) |
| **Food groups** |  |  |  |  |  |
| Offals (g/d) | 0.7 (4.3) | <0.001 | 3.8 (10.8) | <0.001 | 2.3 (8.5) |
| Savoury biscuits (g/d) | 1.9 (4.3) | 0.172 | 1.5 (4.2) | 0.172 | 1.7 (4.3) |
| Cookies (g/d) | 10.5 (18.4) | <0.001 | 5.5 (11.3) | <0.001 | 7.8 (15.3) |
| Alcoholic beverages (ml/d) | 93.2 (141.1) | <0.001 | 149.2 (182.8) | <0.001 | 122.4 (166.6) |
| Hot beverages (ml/d) | 291.1 (249.1) | <0.001 | 548.4 (339.4) | <0.001 | 425.3 (326.0) |
| Breakfast cereals (g/d) | 8.7 (21.2) | 0.001 | 4.7 (14.6) | 0.001 | 6.6 (18.2) |
| Ham, bacon and sausages (g/d) | 27.1 (29.2) | <0.001 | 36.4 (32.6) | <0.001 | 32.0 (31.4) |
| Compotes, fruits in syrup (g/d) | 14.4 (30.9) | 0.566 | 13.3 (26.8) | 0.566 | 13.9 (28.8) |
| Condiments (g/d) | 1.4 (1.5) | <0.001 | 3.3 (3.2) | <0.001 | 2.4 (2.7) |
| Milk-based desserts (g/d) | 46.3 (55.6) | <0.001 | 34.5 (42.1) | <0.001 | 40.1 (49.4) |
| Cheese (g/d) | 24 (22.9) | <0.001 | 41.4 (36.6) | <0.001 | 33.1 (32.0) |
| Fruits (g/d) | 70.4 (72.4) | <0.001 | 180 (153.8) | <0.001 | 127.6 (133.6) |
| Dried fruits (g/d) | 0.4 (2.9) | 0.001 | 1.2 (3.8) | 0.001 | 0.8 (3.4) |
| Nuts (g/d) | 1.8 (4.9) | <0.001 | 4.2 (8.9) | <0.001 | 3.1 (7.3) |
| Juice and nectar (g/d) | 78.5 (96.8) | 0.098 | 66.1 (128.5) | 0.098 | 72.1 (114.6) |
| Milk (ml/d) | 46.4 (92.2) | <0.001 | 11.2 (43.1) | <0.001 | 28 (73.1) |
| Vegetables (g/d) | 69.3 (59.1) | <0.001 | 143.6 (89.3) | <0.001 | 108.1 (84.9) |
| Legumes (g/d) | 5.8 (13.8) | <0.001 | 14 (27.6) | <0.001 | 10.1 (22.5) |
| Fats and oils (g/d) | 8.2 (7.8) | <0.001 | 21.8 (15.2) | <0.001 | 15.3 (14.0) |
| Eggs (g/d) | 12 (19.7) | <0.001 | 17.4 (20.8) | <0.001 | 14.8 (20.5) |
| Bread and rusk (g/d) | 57.1 (41.6) | <0.001 | 121.6 (74.8) | <0.001 | 90.8 (69.2) |
| Pasta (g/d) | 45 (47.5) | <0.001 | 29.7 (33.0) | <0.001 | 37 (41.3) |
| Cakes (g/d) | 52.1 (49.8) | <0.001 | 37.7 (43.7) | <0.001 | 44.6 (47.3) |
| Pizzas and quiches (g/d) | 50.6 (60.5) | <0.001 | 29.6 (44.3) | <0.001 | 39.6 (53.7) |
| Mixed dishes (g/d) | 154.2 (100.5) | <0.001 | 114.2 (92.7) | <0.001 | 133.4 (98.5) |
| Fish and shellfish (g/d) | 20.1 (37.2) | <0.001 | 28.7 (29.4) | <0.001 | 24.6 (33.7) |
| Potatoes (g/d) | 34.5 (38.1) | <0.001 | 49.3 (48.6) | <0.001 | 42.3 (44.5) |
| Sugars and sweets (g/d) | 21.9 (20.0) | <0.001 | 28 (25.8) | <0.001 | 25.1 (23.4) |
| Rice and semolina (g/d) | 30.7 (43.6) | 0.749 | 29.8 (43.7) | 0.749 | 30.2 (43.7) |
| Sandwiches (g/d) | 35.2 (44.0) | <0.001 | 8.9 (19.3) | <0.001 | 21.5 (36.0) |
| Sauces and salad dressings (g/d) | 10.7 (12.1) | <0.001 | 18.5 (14.9) | <0.001 | 14.8 (14.2) |
| Soft drinks (ml/d) | 183.8 (260.1) | <0.001 | 45.8 (114.4) | <0.001 | 111.9 (209.6) |
| Soups (g/d) | 37.2 (78.2) | <0.001 | 84.9 (116.1) | <0.001 | 62.1 (102.6) |
| Dairy products (g/d) | 51.9 (59.8) | <0.001 | 71.5 (67.7) | <0.001 | 62.1 (64.8) |
| Meat (g/d) | 32.9 (34.8) | <0.001 | 41.5 (36.4) | <0.001 | 37.4 (35.9) |
| Pastries (g/d) | 20.4 (27.8) | <0.001 | 7.9 (13.8) | <0.001 | 13.8 (22.5) |
| Poultry (g/d) | 22.7 (27.1) | 0.003 | 28.7 (33.6) | 0.003 | 25.8 (30.8) |
| **Nutrients (unit/day)** |  |  |  |  |  |
| Energy (kcal/d) | 2090.2 (469.9) | <0.001 | 2252.4 (555.9) | <0.001 | 2174.8 (522.8) |
| Water (g/d) | 1847.2 (614.1) | <0.001 | 2257.3 (612.5) | <0.001 | 2061.1 (646.6) |
| Protein (g/d) | 76.1 (21.8) | <0.001 | 86.7 (25.0) | <0.001 | 81.6 (24.1) |
| Carbohydrate (g/d) | 235.4 (57.7) | 0.161 | 229.4 (71.9) | 0.161 | 232.3 (65.6) |
| Sugars (g/d) | 98.1 (37.7) | <0.001 | 87.7 (35.8) | <0.001 | 92.7 (37.1) |
| Total polyols (g/d) | 1 (1.4) | <0.001 | 1.4 (1.4) | <0.001 | 1.2 (1.4) |
| Dietary fiber (g/d) | 17.9 (5.0) | <0.001 | 23.2 (7.0) | <0.001 | 20.7 (6.7) |
| Total fat (g/d) | 83 (22.7) | <0.001 | 92 (26.0) | <0.001 | 87.7 (24.9) |
| SFA (g/d) | 35 (10.6) | 0.001 | 37.4 (12.0) | 0.001 | 36.2 (11.4) |
| MUFA (g/d) | 28.9 (8.9) | <0.001 | 33 (10.9) | <0.001 | 31 (10.2) |
| PUFA (g/d) | 10.6 (3.9) | <0.001 | 12.4 (4.9) | <0.001 | 11.5 (4.5) |
| Cholesterol (mg/d) | 314 (126.6) | <0.001 | 354.9 (129.3) | <0.001 | 335.3 (129.6) |
| Alcohol (g/d) | 7.9 (11.4) | <0.001 | 14.6 (18.1) | <0.001 | 11.4 (15.6) |
| Magnesium (mg/d) | 269.1 (69.7) | <0.001 | 357.6 (165.4) | <0.001 | 315.2 (136.2) |
| Calcium (mg/d) | 844.8 (278.0) | 0.005 | 902.8 (342.3) | 0.005 | 875.1 (314.5) |
| Vitamin B12 (µg/d) | 4.3 (2.6) | <0.001 | 6.2 (5.7) | <0.001 | 5.3 (4.6) |
| **Diet quality score** |  |  |  |  |  |
| NRF9.3 score | 622.8 (95.4) | <0.001 | 717.5 (71.7) | <0.001 | 672.2 (96.3) |
| **IGQ Scores** |  |  |  |  |  |
| Global IGQ score | 13.6 (12.5) | <0.001 | 10.2 (9.4) | <0.001 | 11.9 (11.2) |
| Difficult gas evacuation | 8.2 (17.0) | 0.119 | 6.6 (13.4) | 0.119 | 7.3 (15.2) |
| Stomach rumbling | 14.4 (19.3) | <0.001 | 9.8 (15.6) | <0.001 | 12.0 (17.6) |
| Bad breath | 13.2 (17.5) | <0.001 | 8.5 (13.6) | <0.001 | 10.8 (15.8) |
| Belching | 12.8 (17.0) | <0.001 | 8.4 (13.7) | <0.001 | 10.5 (15.5) |
| Flatulence | 18.4 (18.1) | 0.338 | 17.3 (16.8) | 0.338 | 17.8 (17.4) |
| Bloating | 14.6 (17.9) | <0.001 | 10.8 (15.0) | <0.001 | 12.7 (16.6) |
| N: number of people  SD: standard deviation |  |  |  |  |  |
| P-value based on V-test |  |  |  |  |  |
| SFA: Saturated fatty acids  MUFA: Mono-unsaturated fatty acids  PUFA: Poly-unsaturated fatty acids |  |  |  |  |  |
